# Green Light-Triggerable Chemo-Photothermal Activity of Cytarabine-Loaded Polymer Carbon Dots: Mechanism and Preliminary In Vitro Evaluation

**DOI:** 10.1021/acsami.2c22500

**Published:** 2023-01-23

**Authors:** Grazia M. L. Consoli, Maria Laura Giuffrida, Stefania Zimbone, Loredana Ferreri, Ludovica Maugeri, Michele Palmieri, Cristina Satriano, Giuseppe Forte, Salvatore Petralia

**Affiliations:** † CNR-Institute of Biomolecular Chemistry, Via Paolo Gaifami 18, 95126Catania, Italy; ‡ CIB-Interuniversity Consortium for Biotechnologies, University of Catania, Via Flavia, 23/1, 34148Trieste, Italy; § CNR-Institute of Crystallography, Via Paolo Gaifami 18, 95126Catania, Italy; ∥ Department of Drug Science and Health, University of Catania, Via Santa Sofia 64, 95125Catania, Italy; ⊥ CSEM-Swiss Center for Electronics and Microtechnology, Rue Jaquet-Droz 1, 2002New Chatel, Switzerland; # Department of Chemical Science, University of Catania, Via Santa Sofia 64, 95125Catania, Italy

**Keywords:** carbon dots, photoresponsive polymers, cancer chemo-photothermal therapy, cytarabine, drug delivery, molecular modeling

## Abstract

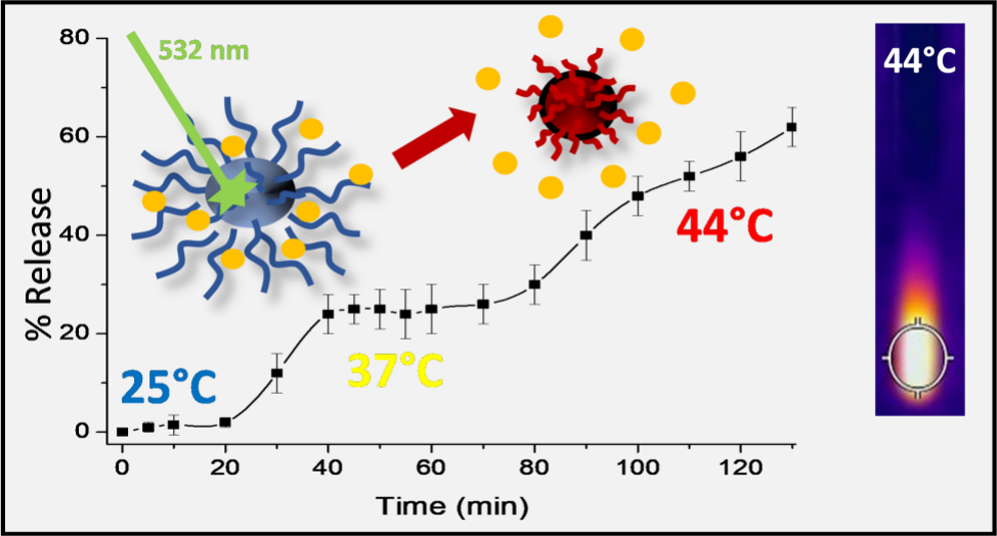

Carbon-based nanostructures are attracting a lot of attention because of their very low toxicity, excellent visible light-triggered optical and photothermal properties, and intriguing applications. Currently, the development of multifunctional carbon-based nanostructures for a synergistic chemo-photothermal approach is a challenging topic for the advancement of cancer treatment. Here, we report an unprecedented example of photoresponsive carbon-based polymer dots (CPDs-PNM) obtained by a one-pot thermal process from poly(*N*-isopropylacrylamide) (PNIPAM) without using organic solvent and additional reagents. The CPDs-PNM nanostructures were characterized by spectroscopic techniques, transmission electron microscopy, and atomic force microscopy. The CPDs-PNM exhibited high photothermal conversion efficiency, lower critical solution temperature (LCST) behavior, and good cytarabine (arabinosyl cytosine, AraC) loading capacity (62.3%). The formation of a CPDs-PNM/AraC adduct and photothermal-controlled drug release, triggered by green light excitation, were demonstrated by spectroscopic techniques, and the drug–polymer interaction and drug release mechanism were well supported by modeling simulation calculations. The cellular uptake of empty and AraC-loaded CPDs-PNM was imaged by confocal laser scanning microscopy. In vitro experiments evidenced that CPDs-PNM did not affect the viability of neuroblastoma cells, while the CPDs-PNM/AraC adduct under light irradiation exhibited significantly higher toxicity than AraC alone by a combined chemo-photothermal effect.

## Introduction

Photothermal and photoluminescent carbon-based nanostructures are a new class of materials widely studied in life science due to their biocompatibility, optical properties, nanosized structure, and high versatility.^1^ They have been investigated for applications as sensors, photocatalysts, bioimaging agents, drug and gene delivery systems, and most recently in chemo-photothermal therapy (chemo-PTT) for cancer treatment.^2−4^ Gold nanostructured materials are the leading nanomaterials used in PTT^5^ and photothermal-triggered drug release.^6^ Other inorganic nanostructures such as iron oxide,^7^ copper sulfide,^8,9^ BiAgOS nanoparticles,^10^ mesoporous organosilica,^11^ titanium carbide derivates,^12^ pegylated-SiO*_x_*/CeO_2_/VO*_x_*,^13^ and cobalt oxide^14^ nanoparticles have also been frequently used in biomedical applications. However, low stability, long-term toxicity, and high cost have drastically limited their diffusion in PTT. To overcome these drawbacks, photothermal-responsive carbon-based nanomaterials such as carbonized polymer dots (CPDs), carbon quantum dots, and graphene oxide have attracted strong interest.^15,16^ CPDs are fascinating materials that, by combining the optical properties of the nanosized carbon dots with the multifunctionality of the polymer, can provide multiresponsive nanostructures for chemo-PTT. The synergistic effect between PTT and chemotherapy is one of the latest therapy methods to cure cancer.^17^ As a noninvasive therapeutic strategy, PTT offers great advantages such as safety, high efficiency, a broad spectrum of action, and light-controlled site-specific drug delivery. PTT employs photoabsorbers to convert photon energy into heat. The light-induced hyperthermia may enhance the cytotoxicity of some chemotherapeutics, with consequent more effective anticancer effects than either therapy alone. Moreover, the delivery of drugs to tumorigenic regions by a photothermal nanocarrier is an appealing approach for a safer therapy. Indeed, hyperthermia and drug release confined to irradiated areas can reduce undesired side effects of conventional chemotherapy. In this scenario, poly(*N*-isopropylacrylamide) (PNIPAM) is a photothermal and photoresponsive material, which thanks to its chemical stability, low toxicity, and temperature- and pH-responsivity is largely used for biomedical applications such as drug delivery, sensing, and catalyst agent.^16,18^ Basically, at temperatures below the lower critical solution temperature (LCST), the PNIPAM chains adopt a coil-extended water-exposed conformation stabilized by favorable interactions with water molecules. When the temperature increases above the LCST value (30–33 °C), a cooperative transition occurs and PNIPAM chains adopt a more hydrophobic globule conformation. In this collapsed state, water molecules are expelled from the shell, decreasing the amount of hydrogen bonds.^19^ The globule conformation is assumed by the polymer also at acid pH values. Indeed, the protonation of the carbonyl group destabilizes the coil conformation favoring the globule conformation.^20^ Light- and pH-induced conformational changes are key to the reversible drug loading and release mechanisms of PNIPAM. Nontoxic engineered nanomaterials, which function as both drug nanocarriers and photothermal agents, are proving to be one of the vigorously studied multimodal ploys to enhance the anticancer effect of many drugs.

Cytarabine, also known as 1-β-d-arabinofuranosylcytosine (AraC), is a nucleoside drug whose anticancer mechanism of action has been ascribed to incorporation into replicative DNA. AraC works as an effective antimetabolite agent, by competing with pyrimidine, and induces incompletion or defective ligation during DNA replication, leading to cell death.^21^ Preclinical studies and clinical trials have shown that the entrapment of AraC in a liposomal nanocarrier reduces cytotoxic side effects compared to free drugs.^22^ As far as we know, only a case of hyaluronic acid/AraC-IR820@ZIF-8 nanoparticles for chemo-PTT was reported in the literature.^23^ Recently, we developed photothermal and luminescent nanosized CPDs based on PNIPAM (CPDs-PNM) by an innovative one-pot method^24^ and demonstrated their photocontrollable ability to load and release curcumin selected as a drug model. Here, we report a more in-depth characterization of the CDP-PNM structure and optical properties and the investigation of the potential of CPDs-PNM as a luminescent and photothermal AraC nanocarrier for chemo-PTT. Molecular modeling studies were performed to better understand the mechanism of drug loading and release. The Uptake of AraC into neuroblastoma cells was demonstrated, and biological assays were carried out to evaluate the light-triggered capability of CPDs-PNM to enhance the AraC cytotoxic effect.

## Results and Discussion

**Scheme 1:**
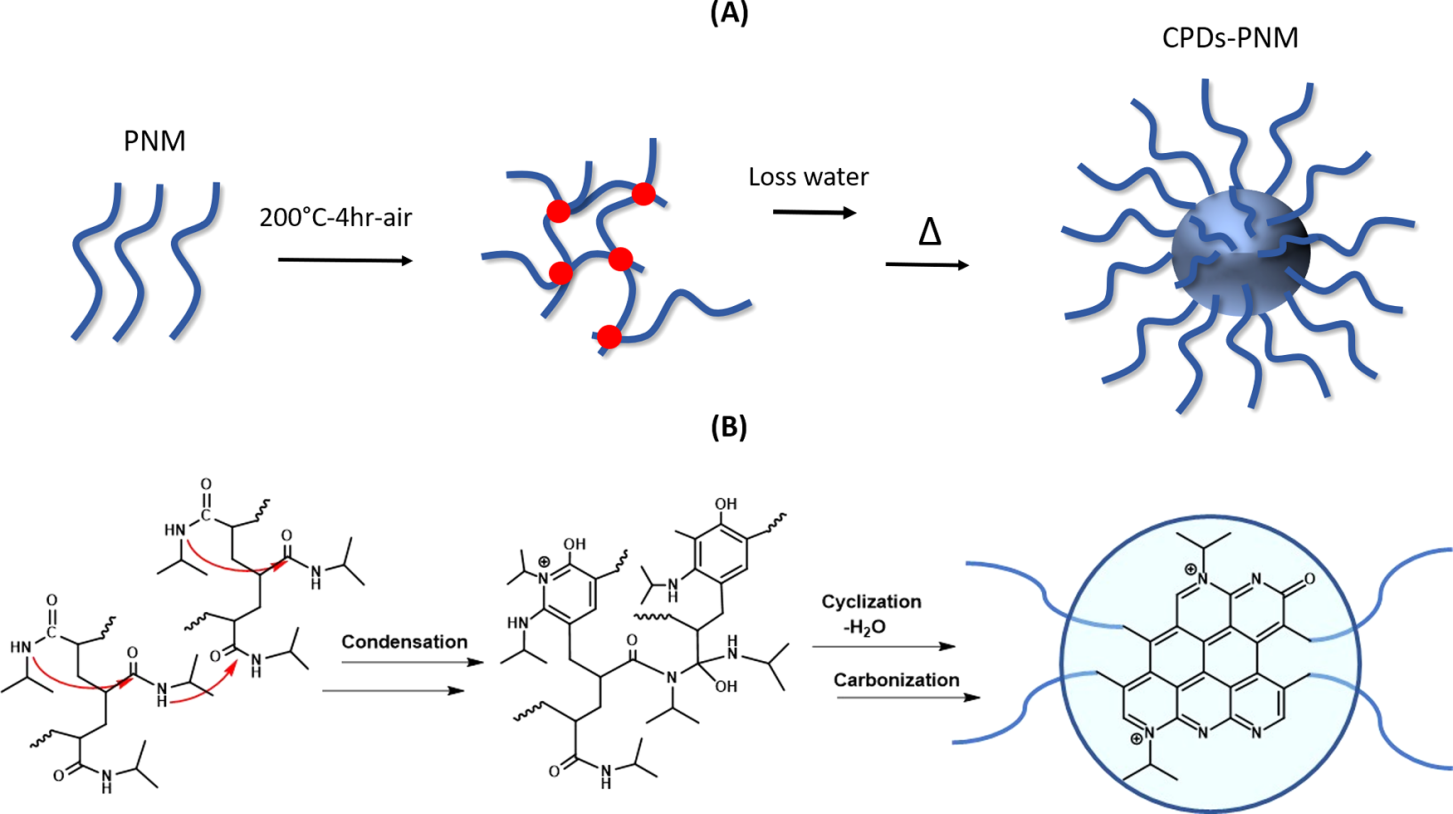
CPDs-PNM: (A) Schematic Representation of the CPDs/PNM Formation Process and (B) Proposed Mechanism for the Formation of a Possible CPDs-PNM Structure

Carbon polymerized dots with poly-*N*-isopropylacrylamide pendants (CPDs-PNM) were prepared from poly(*N*-isopropylacrylamide) (PNIPAM) using an innovative one-pot method recently developed in our laboratories^24^ (procedure reported in the Methods section). Heating of the precursor polymer PNIPAM for 4 h at 200 °C in air provided CPDs-PNM, as depicted in Scheme 1A. Interestingly, the thermal reaction was carried out without using solvents, oxidizing agents, acids, and instrumentation (i.e., microwave, autoclave, etc.) currently used for the preparation of similar carbon polymer dots. The cross-linking mechanism proposed for the formation of the carbon core is based on a chain condensation-cyclization process, followed by water loss and aromatization, as depicted in Scheme 1B.

The proposed cross-linking mechanism was investigated by different techniques. UV–vis optical absorption measurements at various carbonization process times (0, 20, 60, 120, and 240 min at 200 °C) showed that the cross-linking occurred during the first 20 min of the process (Figure S1). After 20 min of heating, the resulting uncolored purified dispersion showed an optical absorption spectrum with the typical band for the amide groups (280 nm). No absorption bands related to π–π* (270 nm) and n−π* (360 nm) transitions, indicative of carbon core formation, were observed. The carbon core formation was spectroscopically evident after 60 min of heating (Figure S1). The subsequential loss of water is crucial for the chain cross-linking phase before the carbonization process, as corroborated by the failure of the hydrothermal carbonization performed at 200 °C for 4 h under microwave treatment.

The proposed condensation/aromatization mechanism was supported by NMR investigation. The ^1^H NMR spectrum of CPDs-PNM prepared by heating at 200 °C for 4 h showed CH_3_ (1.13 ppm) and CH (3.99 pm) resonances relative to the isopropyl groups of the polymer pendants. By increasing the temperature from 200 to 300 °C, novel CH_3_ (1.28 ppm) and CH (4.85 ppm) signals, consistent with isopropyl groups bonded to aromatic nitrogen atoms, appeared in the proton spectrum. The intensity of these signals increased by prolonging the heating time from 30 min to 8 h, while the signals of the polymeric pendants decreased (Figure S2). Thus, it can be assumed that higher temperatures and longer heating times favor the condensation/aromatization mechanism.

**Figure 1:**
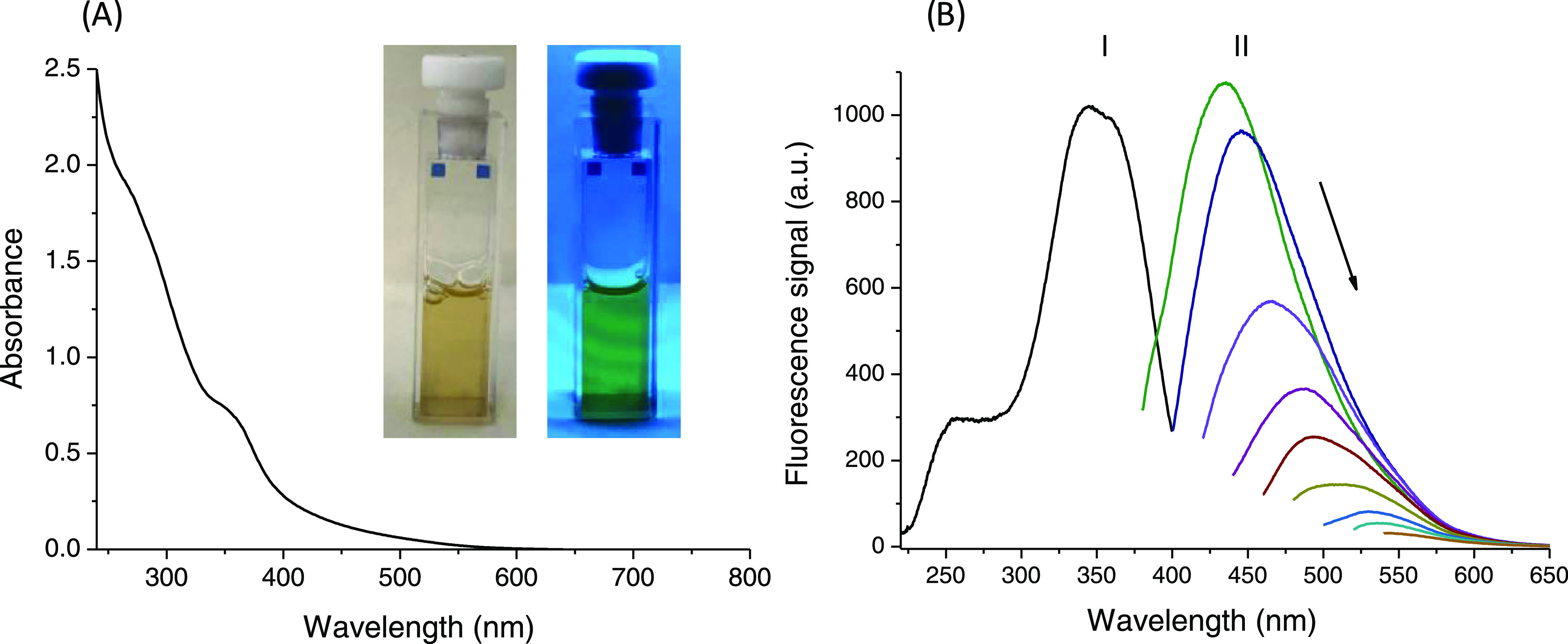
CPDs-PNM dispersion in water: (A) optical absorption spectrum, inset pictures of CPDs-PNM under environment light (left) and under a 254 nm UV lamp (right) and (B) excitation spectrum (line I) and emission fluorescence spectra (line II) at various excitation wavelengths (360, 380, 400, 420, 440, 460, 480, 500, and 520 nm).

UV–vis optical absorption spectra of CPDs-PNM exhibited the typical absorption bands for aromatic nitrogen-doped carbonized polymer dots. The spectra showed a band centered at 275 nm referred to as π–π* transition that originates from sp^2^ carbon and a well-defined band centered at 360 nm, related to the n−π* transition generated from C=C and C=N bonds (Figure 1A). Similarly, the excitation spectra reported diagnostic bands at 360 and 260 nm (Figure 1B line I). Figure 1B line II depicts the fluorescence emission spectrum for CPDs-PNM at various excitation wavelengths from 360 to 520 nm. With increasing excitation wavelength, the CPDs-PNM water dispersion showed an excitation-dependent emission typical for carbon-based dots.

**Figure 2:**
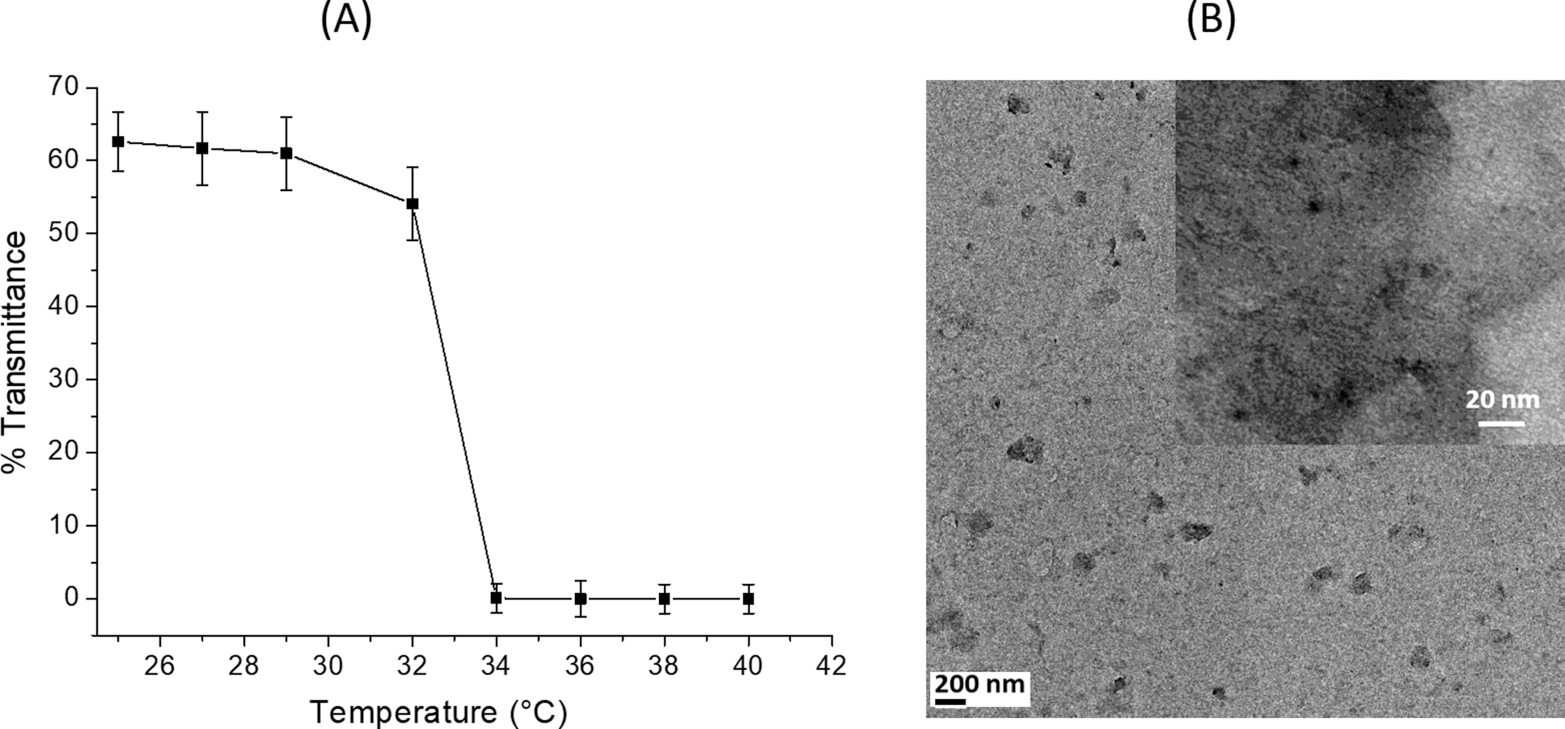
CPDs-PNM: (A) LCST behavior: transmittance measurements at various temperatures and (B) representative TEM images.

The optical transmittance measurements recorded for the CPDs-PNM at temperatures in the range from 25 to 40 °C showed a typical LCST value of about 32–33 °C. Indeed, the transmittance values decreased from 60 to 1–5% at temperatures above the LCST value (Figure 2A). This behavior confirmed the PNIPAM structure transition from coil-extended to globule conformation at temperatures above the LCST value (32–33 °C).

The morphology of CDPs-PNM was investigated by microscopy techniques. TEM images showed the presence of large carbon nanostructures with sizes ranging from 80 to 120 nm (Figure 2B) relative to the whole CPDs-PNM structure (core + pendants), and spherical carbon cores with diameters around 10 nm (Figure 2B inset).

**Figure 3:**
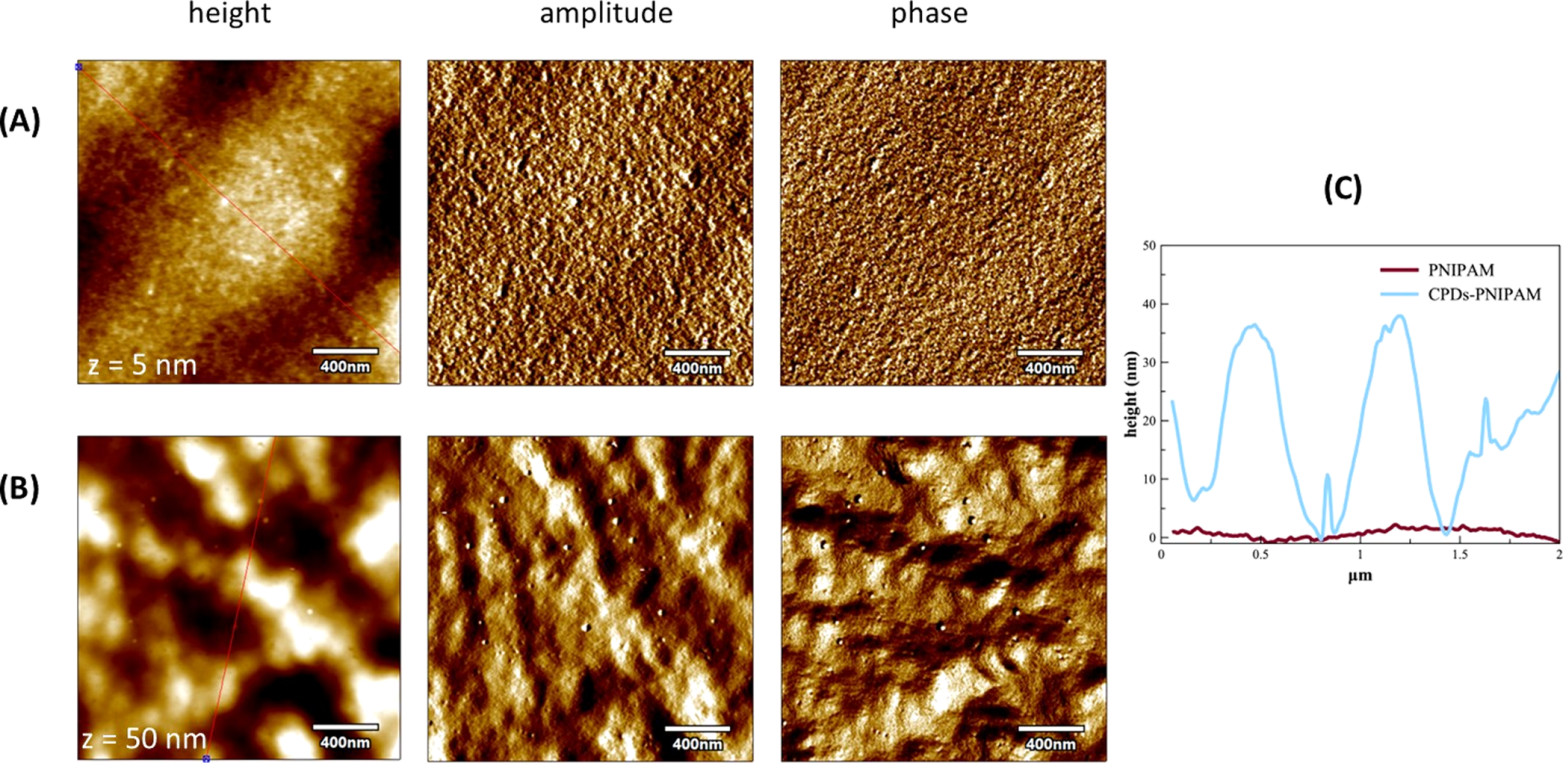
Representative AFM images of the height, amplitude, and phase for PNIPAM (A) and CPDs-PNM (B) at pH 7.4 and the corresponding section analysis curves (C).

AFM analyses of the PNIPAM (Figure 3A) and CPDs-PNM (Figure 3B) samples at pH 7.4 showed a clear roughening of the polymer due to the thermal treatment, as especially evident from the amplitude and phase images. Specifically, bundles of polymer chains with an average peak-to-valley distance value of ∼45 nm were evident in the CPDs-PNM sample (Figure 3C) that also showed small spherical nanoparticles of the CPD core with an average size of 7.2 ± 0.5 nm.

**Figure 4:**
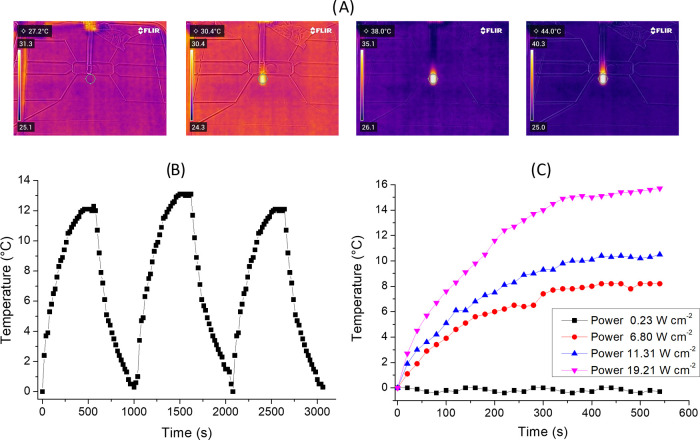
(A) Photothermal cycles for CPDs-PNM; (B) representative thermophotographs of the CPDs-PNM dispersion in water during the photothermal experiments; and (C) photothermal effect of the CPDs-PNM dispersion (100 μL, Abs_532 nm_ = 0.29) at different laser power densities.

To investigate the photothermal properties of CPDs-PNM, an aqueous dispersion (100 mL, Abs_532 nm_ = 0.29) was continuously exposed to a 532 nm laser (200 mW power). The temperature changes were monitored by a thermal camera (Figure 4A). When the temperature of the system reached the maximum value of about 38 °C (temperature difference = *T*_max_ – *T*_environment_ = 12 °C), the laser was switched off, and the temperature change during cooling was monitored to confirm the heat transfer of the system (Figure 4B). A photothermal conversion efficiency (η) value of 43.8% was calculated (Supporting Information and Figure S3). To better investigate the correlation between the photothermal activity and the absorbance at 532 nm, we also performed experiments using CPDs-PNM dispersions with different absorbance values. The results reported in Figure S4 indicated temperature increases of about 4.1, 7.5, 11, and 18 °C for *A*_532 nm_ = 0.043, 0.08, 0.11, and 0.43, respectively.

The cycles were repeated many times to confirm the reversible process (Figure 4B reports three representative photothermal cycles). The power-dependent behavior was confirmed by the experiments performed with different laser powers of 4, 12, and 339 mW, and the temperature difference values recorded were about 0, 8, 10, and 16 °C, respectively (Figure 4C).

### CPDs-PNM/AraC Adduct Preparation and Characterization

**Figure 5:**
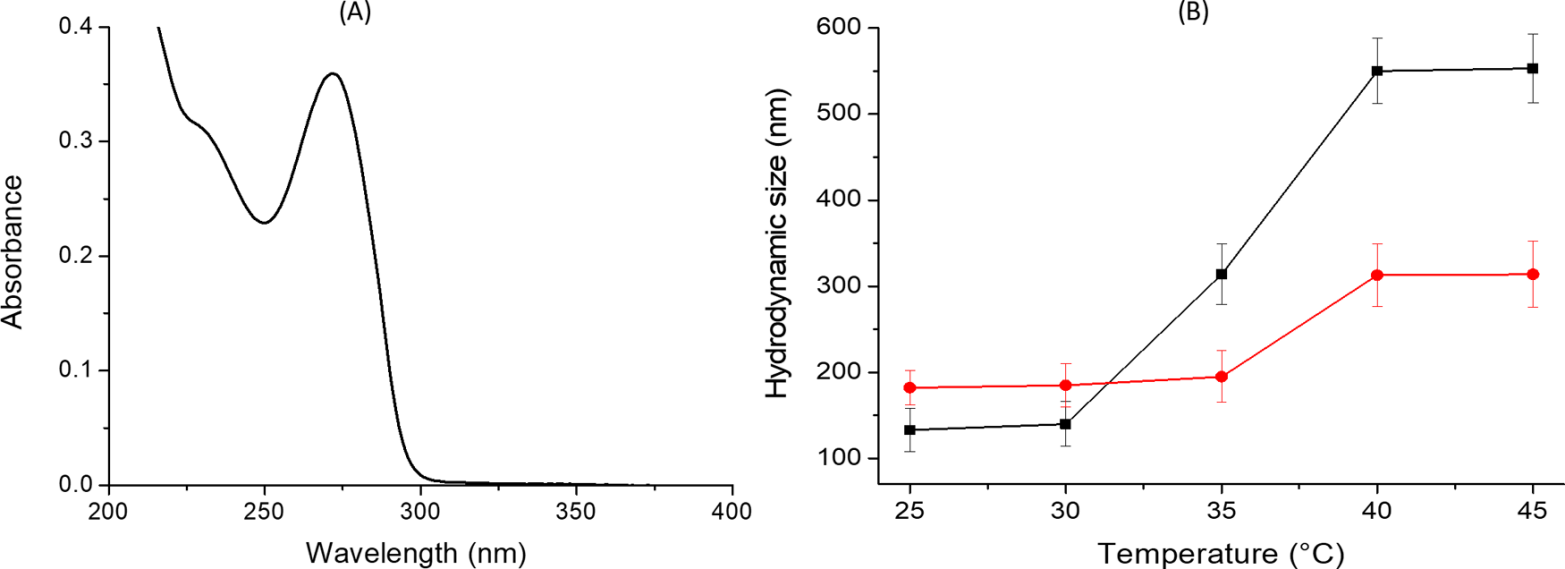
CPDs-PNM/AraC adduct: (A) representative UV–vis optical absorption spectrum and (B) DLS measurements at different temperatures (25, 30, 35, 40, and 45 °C) at pH 7 (black square) and pH 5.0 (red circle).

To load AraC into CPDs-PNM, an excess of AraC (1:3 w/w) was added to the CPDs-PNM colloidal solution, and the mixture was stirred for 48 h. Then, the sample was dialyzed to remove the unentrapped drug. The drug loading capacity percentage was calculated to be 62.3%. The CPDs-PNM/AraC adduct formation was confirmed by spectroscopic and DLS measurements and supported by modeling simulation data. The UV–vis absorption spectrum of CPDs-PNM/AraC (Figure 5A) showed the AraC absorption band at 271 nm and the typical n−π* band of the CPD core at 360 nm. ^1^H NMR spectra displayed a slight upfield shift (Δδ = 0.010–0.013 ppm) of the entrapped drug signals (AraC pyrimidine and sugar CH protons, Figure S5).

Dynamic light scattering measurements evidenced that at pH 7, the CPDs-PNM/AraC nanoparticles possess a mean hydrodynamic diameter (intensity %) of around 133–140 nm at 25–30 °C. This value increased to 314, 550, and 553 nm at 35, 40, and 45 °C, respectively. At higher temperatures, the polymer undergoes a conformational variation that provides aggregates with a more hydrophobic surface that tends to further aggregate in a polar solvent such as water. Indeed, as reported in the literature, during the transition, water molecules are expelled from the polymer shell (coil conformation), the amount of hydrogen bonds decreases, and the hydrophobicity drastically increases (globule conformation).^19^ At acid conditions (pH 5.0), according to the drastic destabilization of the coil conformation, the transition to the more hydrophobic globule conformation is favored,^20^ and at 25–30 °C, a mean hydrodynamic diameter of 182–185 nm higher than the one at neutral pH was observed for the CPDs-PNM/AraC adduct. A slighter increase to 195, 313, and 316 nm was instead measured at 35, 40, and 45 °C, respectively (Figure 5B).

**Figure 6:**
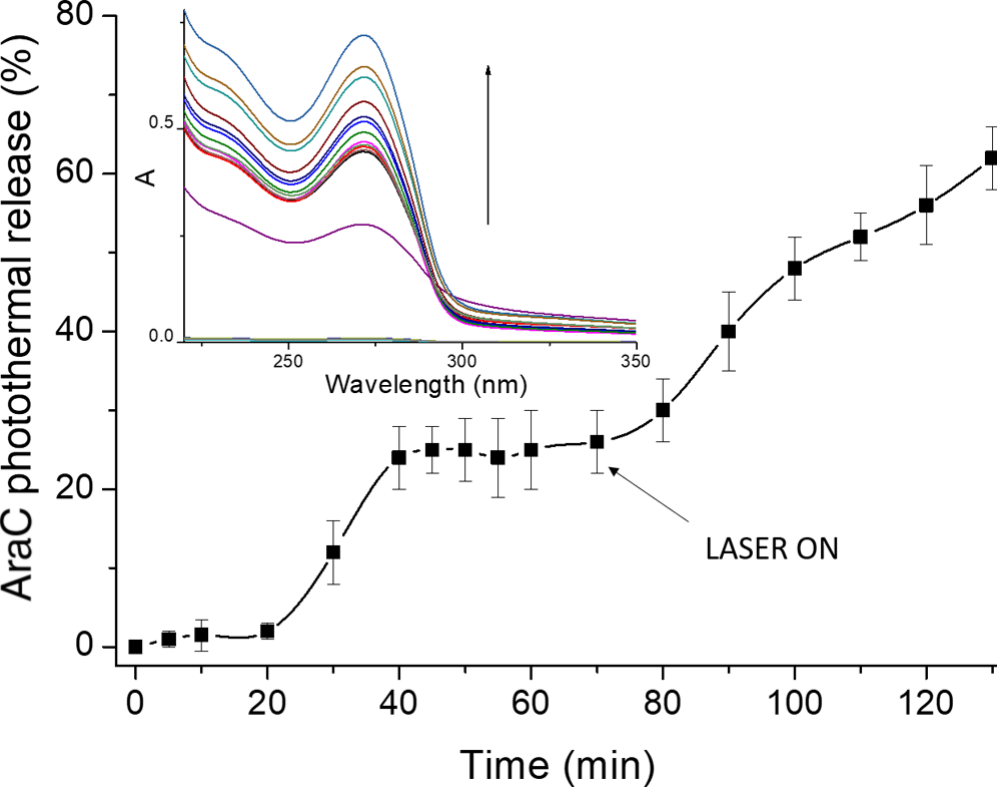
Photothermal-triggered release of AraC from the CPDs-PNM/AraC dispersion (*A*_532 nm_ = 0.07); the inset shows the diagnostic absorption band of AraC in solution upon incubation at 25 and 37 °C and green light laser exposition.

To assess the photothermal-induced release of the drug from the CPDs-PNM/AraC adduct, the dispersion was incubated for 20 min at room temperature in dark conditions, and the absorbance measurements at various incubation times (0, 5, 10, and 20 min) were recorded. Then, the temperature was regulated to 37 °C. The absorbance spectra indicated the absence of AraC release at room temperature, while a boost release of the drug after 10 min (about 11%) and 20 min (about 22%) of incubation at 37 °C was observed (Figure 6).

When the temperature was increased to a maximum value of about 44 ± 2 °C by a few minutes of irradiation with a CW laser (532 nm–19.21 W/cm^2^), the absorption measurements showed a photothermal-induced drug release. Under green light stimulation, at various exposition times from 70 to 130 min, an effective drug release with a rate of about 0.8 ± 0.05%/min was recorded.

Modeling investigations suggested that the drug loading process occurs with the formation of stable H-bonds between AraC and PNIPAM. To gain information about the AraC-AraC and PNIPAM-AraC interaction energy binding values were calculated by modeling simulations. First, the interactions between 2, 4, and 6 molecules of AraC were measured by the calculated binding energies of AraC_i_ in the water solvent. These values are referred to the difference between the electronic energy of the cluster and the electronic energy of the single molecule.^a^ Binding energy values of −17.41, −41.69, and −55.35 kcal/mol were found for AraC clusters with 2, 4, and 6 molecules, respectively. The energy values became more negative as the number of molecules increased, indicating that very stable clusters are formed in water mainly due to the formation of intermolecular hydrogen bonds between the cytosine nucleobases and between the arabinose residues (Figure S6). The data showed that an extended hydrogen bond network is formed among the AraC molecules. In particular, each cytosine base can interact with the three closest bases, whereas each sugar residue is H-bonded with two adjacent residues of arabinose. Similarly, the binding energy values for the PNIPAM-AraC_*i*_ adducts after MD simulations and further optimization were calculated^b^. The data showed for the adducts with 1, 2, and 4 AraC molecules binding energy values of −15.59 kcal/mol for PNIPAM-AraC_1_, −17.63 kcal/mol for PNIPAM-AraC_2_, and −19.34 kcal/mol for PNIPAM-AraC_4_ (all simulations were replicated three times, Table S1). These values clearly indicate an augmented and favorable interaction with increasing AraC units (4 > 2 > 1 unit).

**Figure 7:**
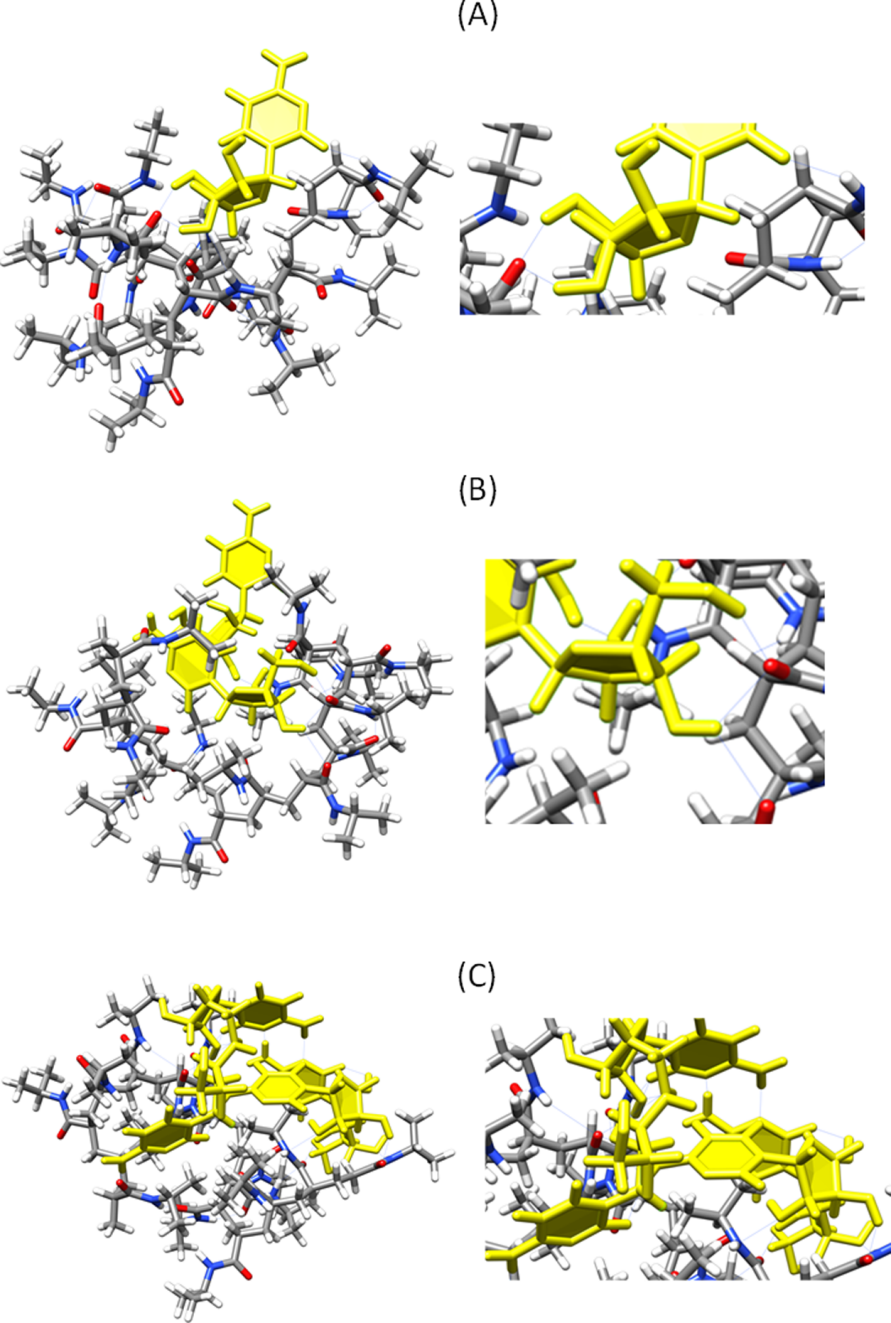
Modeling of the PNIPAM-AraC_*i*_ adduct for (A) *i* = 1; (B) *i* = 2, and (C) *i* = 4 AraC units. The blue line shows the formed hydrogen bonds.

Additional modeling investigations showed that for PNIPAM-AraC_*i*_ adducts with *i* = 2 and 4, the drug molecules are rearranged in different orders compared to the AraC-AraC clusters. Indeed, the intermolecular H-bond network becomes less stable and new H-bonds between AraC and PNIPAM, which is in the globule form, are formed. Figure 7 depicts the H-bond interactions between the hydroxyl group of arabinose units with the amidic groups of PNIPAM for the adducts with 1, 2, and 4 AraC units.

**Figure 8:**
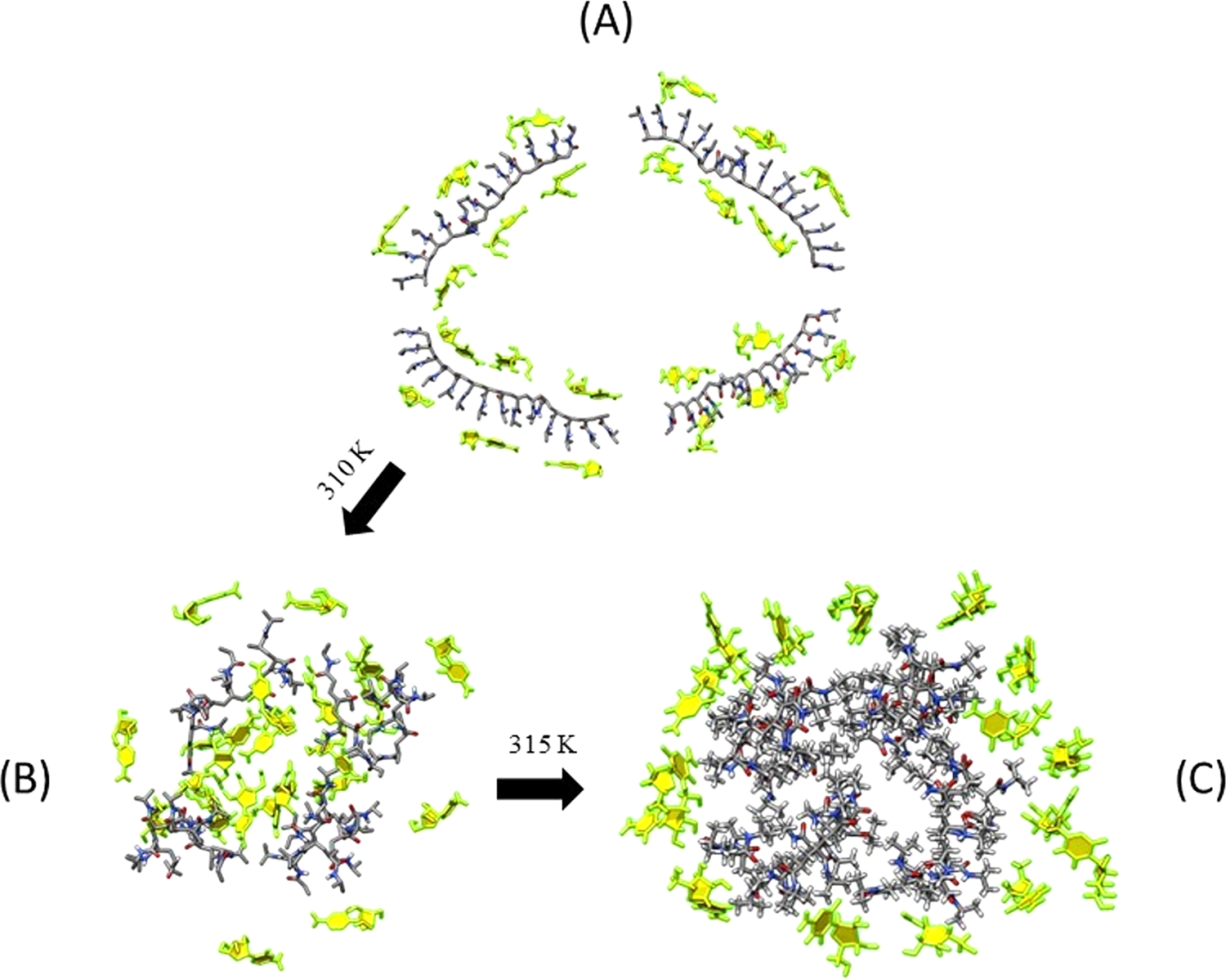
Two-step mechanism for AraC release based on the PNIPAM coil-to-globule structure transition: (A) PNIPAM coil structure at 298 K; (B) PNIPAM globular structure at 310 K with the release of about 30% of AraC; and (C) the remaining AraC molecules are expelled with the formation of PNIPAM globule aggregates at 315 K.

All these findings well corroborate with the photothermal kinetics release of AraC reported in Figure 6. In particular, the massive release observed at 310 K (37 °C) can be mainly attributed to the AraC molecules, which interact weakly with PNIPAM. Increasing kinetic energy to 315 K (42 °C) gives rise to a further release of the AraC molecules that form more effective hydrogen bonds with the PNIPAM. To corroborate these data, DLS measurements at different temperatures reported in Figure 5 showed that CPDs-PNM-AraC_*i*_ adducts tend to aggregate as the temperature increases. Like the mechanism underlying the coil-to-globule transition,^25,26^ the aggregation releases the remaining tightly bound AraC molecules, giving rise to macroaggregates of PNIPAM, as illustrated in Figure 8.

### Cell Viability Experiments

We previously demonstrated the biocompatibility of CPDs-PNM on the neuronal cellular model SH-SY5Y.^24^ In this paper, we moved on to the evaluation of the efficacy of the CPDs-PNM as a nanocarrier for drug delivery and a novel tool for chemo-PTT. To this end, we investigated the capability of CPDs-PNM to ameliorate the cytotoxic effect of AraC that kills cancer cells by interfering with DNA synthesis.^27^ Currently, AraC is used in high doses and combined with anthracyclines, usually daunorubicin or idarubicin, for the treatment of acute myeloid leukemia (AML).^28^ Complete remission in up to 80% of young patients has been observed; however, several side effects including gastrointestinal disturbances, hepatotoxicity, and neurotoxicity have been reported.^29^ In the last few decades, new formulations have been proposed to limit the side effects or improve the pharmacokinetics of AraC. It was demonstrated that polymeric carriers can be effective tools to prolong the circulation half-life, promote selective targeting, and provide a controlled release of the loaded drug.^30^

**Figure 9:**
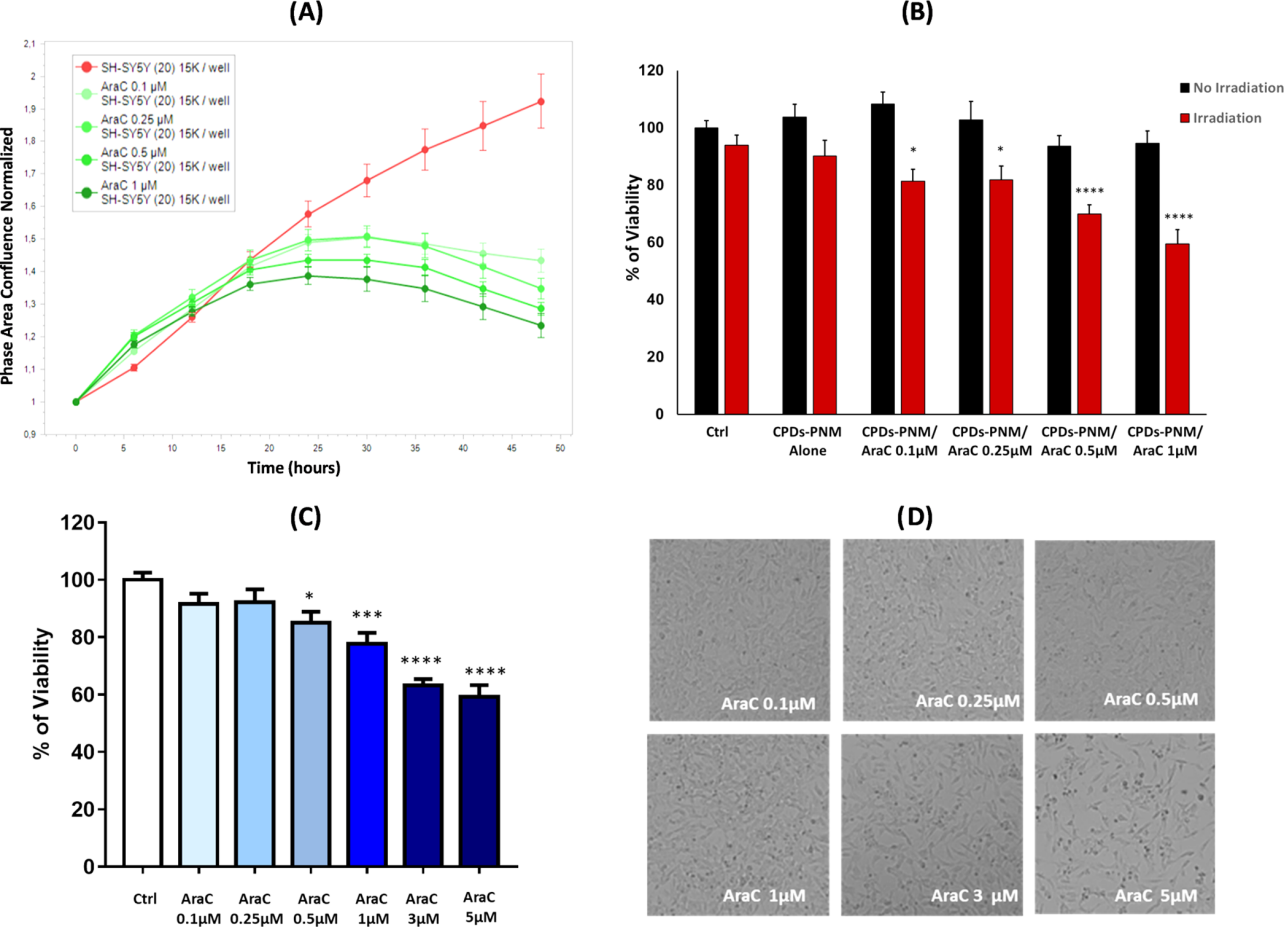
(A) Antiproliferative response in neuroblastoma SH-SY5Y cells to increasing concentrations of AraC (0.1–1 μM) as quantified by the Incucyte SX1 Live-Cell Analysis System. The readout of cellular growth was assessed every 6 h over 24 h by Cell-by-Cell Analysis Software. Values for each timepoint represent means ± SEM of 3 replicates and are normalized to control wells. (B) MTT analysis of the neuroblastoma cell lines treated with CDPs-PNM (0.2 mg/mL) alone or loaded with increasing concentrations of AraC (0.1–1 μM) and exposed or not for 4 min to light (λ = 532 nm). Cell viability was assessed after 24 h of treatment. Bars represent means ± SEM of three independent experiments with *n* = 3 each. ****P < 0.0001, *P < 0.05 versus Ctrl w/o irradiation by one-way ANOVA + Tukey test. (C) Dose–response effect of AraC on SH-SY5Y, MTT analysis of neuroblastoma cell lines, SH-SY5Y, treated with increasing concentrations of AraC (0.1–5 μM) for 24 h. Bars represent means ± SEM of three independent experiments with *n* = 3 each. ****P < 0.0001, ***P < 0.001, *P < 0.05 versus Ctrl by one-way ANOVA + Tukey test. (D) Representative optical images of neuroblastoma cells after 24 h exposure with AraC (0.1–5 μM).

To study the effect of the CPDs-PNM/AraC adduct on cancer cell viability, we first tested the antiproliferative effect of AraC on SH-SY5Y neuroblastoma cells by the Incucyte Live-Cell Analysis System. We applied a dose, ranging from 0.1 to 1 μM, of AraC for 48 h, and by Cell-by-Cell Analysis Software, we obtained a readout of cytotoxicity over time in a label-free manner and automatically analyzed it in living cells, within an incubator. As expected, the time course revealed a concentration-dependent effect of AraC, which became more evident after 18–20 h of treatment, when cells exposed to the drug stopped proliferating compared to untreated cells (Figure 9A). To optimize the use of the newly synthesized CPDs-PNM, we also performed an MTT assay on the neuroblastoma cell lines exposed to higher doses of AraC for 24 h. We found that even if statistically different from the control, 1 μM AraC produced only 20% of toxicity, while 5 μM AraC provided a 40% decrease in cell viability (Figure 9C) as confirmed by the affected density of the cells (Figure 9D).

Based on these findings, to explore whether the delivery by CPDs-PNM can improve the AraC activity, we studied the effect of CPDs-PNM/AraC samples containing a constant concentration of CPDs-PNM (0.2 mg/mL) and subtoxic amounts of loaded AraC (0.1–1 μM range). MTT assays were performed on (i) untreated cells (control), (ii) cells treated with CPDs-PNM, and (iii) cells treated with CPDs-PNM/AraC adducts, without irradiation and irradiation by a CW laser 532 nm. The MTT assay after 24 h of incubation showed no significant effect on cell viability for all nonirradiated samples and irradiated CPDs-PNM (Figure S7). Interestingly, an effective dose–response cytotoxic effect was instead evident on irradiated cancer cells treated with CPDs-PNM/AraC (Figure 9B). The cytotoxic effect is consistent with the light-induced CPDs-PNM conformational change and consequent drug release. As a confirmation, without irradiation, the drug is entrapped in the nanocarrier, and the CPDs-PNM/AraC adduct (CPDs-PNM/AraC 1 μM, Figure 9B) results to be less cytotoxic than AraC alone (AraC 1 μM, Figure 9C). Noteworthy, the cytotoxic effect of the CPDs-PNM/AraC adduct after light exposure was higher than the one of AraC alone (see dose–response in Figure 9C,D). AraC at 1 μM concentration affected cell viability by less than 20%, whereas 40% of cell death was observed for the same concentration of AraC delivered by the CPDs-PNM nanocarrier, after green light irradiation. A higher amount of AraC alone (5 μM concentration) was required to induce 40% of cell death (Figure 9C). Irradiation (green light for 4 min) of the cells treated with CPDs-PNM and the CPDs-PNM/AraC adduct enhanced the cell temperature to 43 °C, as evidenced by a thermal camera. Representative thermographs and the related temperature data during green light irradiation of CPDs-PNM on a 96-well plate are reported in Figure S8. It is plausible to think that light-induced hyperthermia may contribute to the higher cell damage effect of the CPDs-PNM-delivered AraC. It is known that hyperthermia can enhance the effect of anticancer drugs, and the hyperthermia–chemotherapy combination improves the treatment of advanced and recurrent cancers.^30^

### Cellular Uptake Experiments

**Figure 10:**
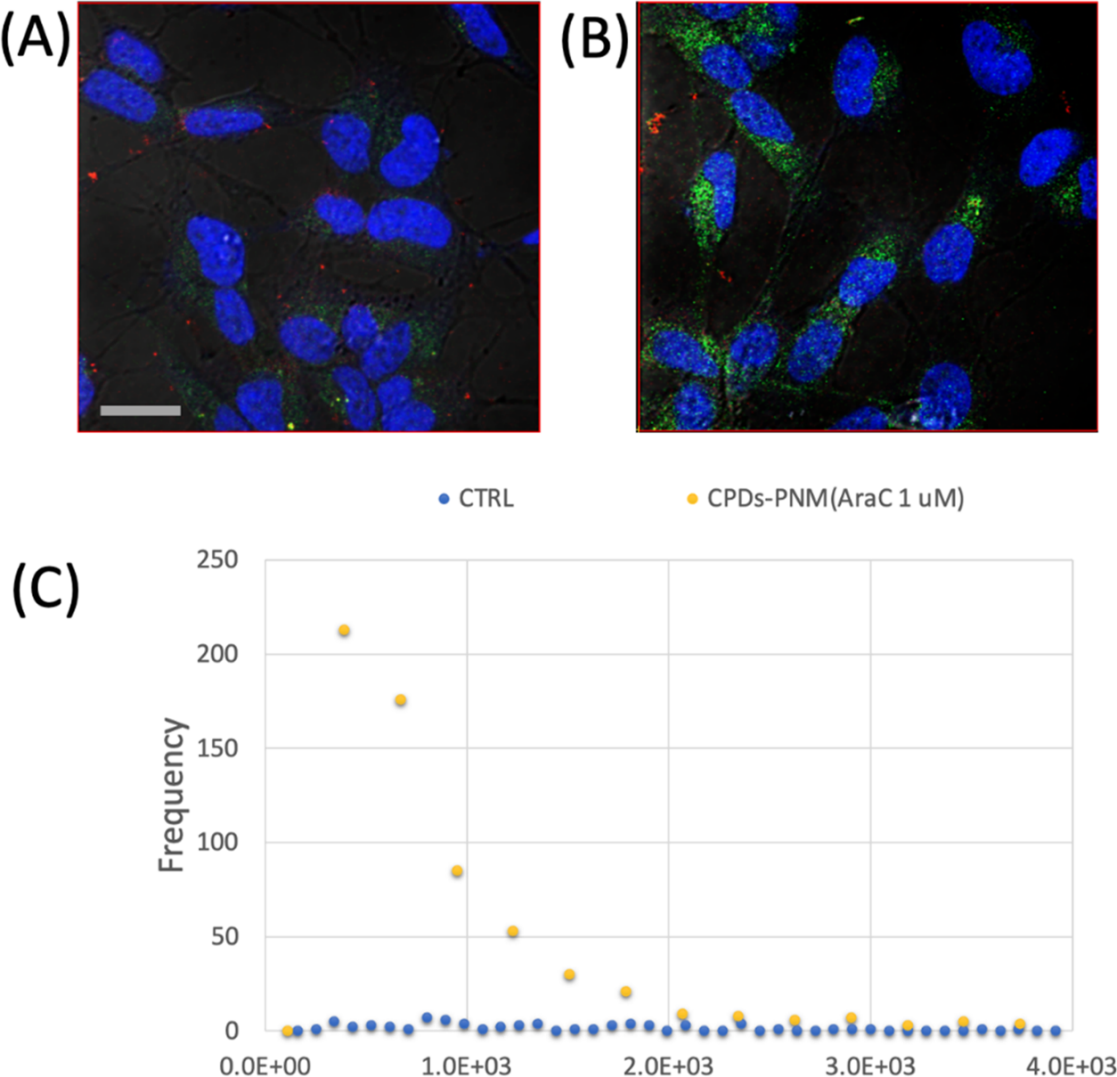
Representative laser scanning microscopy micrographs with merged channels of the optical bright field (gray) and emission in blue (λ_ex/em_ = 405/410–430 nm), green (λ_ex/em_ = 488/500–530 nm), and red (λ_ex/em_ = 633/650–700 nm) for neuroblastoma SH-SY5Y cells untreated (A) or treated (B) with CPDs-PNM/AraC (1 μM). Scale bar = 20 μm. (C) Histograms for the quantitative analysis of the green fluorescence.

To evaluate the potential of the CPDs-PNM as a drug delivery system, cellular uptake experiments were performed on neuroblastoma SH-SY5Y cells. The confocal microscopy analysis of untreated cells, used as a negative control, showed no significant emission apart from the cell autofluorescence (Figure 10A). Green staining was instead detected in the cells incubated with the CPDs-PNM/AraC adduct (Figure 10B). This finding evidenced that the luminescent CPDs-PNM penetrate inside the cells. The quantitative assessment of the green fluorescence is reported in the histograms in Figure 10C.

All findings suggest that the higher cell mortality induced by the irradiated CPDs-PNM/AraC adduct (40% cell mortality at AraC 1 μM concentration) compared to the free drug (20% cell mortality at 1 μM concentration) could be reasonably explained by a synergistic contribution of (i) the effective cellular uptake of the drug-loaded nanostructures as demonstrated by confocal microscopy analysis, (ii) intracellular photothermal-induced drug release, and (iii) more drug efficacy by hyperthermia-enhanced cell sensitivity.

## Conclusions

Biocompatible, water-dispersible, and green light photothermal-responsive nanosized carbon polymer dots (CPDs-PNM) were prepared in one step by thermal treatment of photoresponsive PNIPAM. The CPDs-PNM were characterized for physicochemical and photothermal properties as well as drug loading and release capacity. As corroborated by molecular modeling, a green light-induced conformational change controls the release of AraC, selected as a model of the anticancer drug, from the CPDs-PNM. The CDP-PNM/AraC adduct can cross the membrane of neuroblastoma cells and reach the cytoplasm where the irradiated CDPs-PNM release the drug and by photothermal conversion increase the local temperature. A combined chemo-photothermal effect may be involved in the higher in vitro tumor cell mortality induced by the CDP-PNM/AraC adduct compared to the free drug. Easy and green preparation without using organic solvent and additives, biocompatibility, drug delivery capacity, light-controlled drug release, and hyperthermia as an adjuvant to effectively enhance the sensitivity of cancer cells to a chemotherapeutic are features that make the CPDs-PNM appealing in the research of novel photoresponsive nanocarriers for drug delivery and drug–hyperthermia combination.

## Methods

### Chemicals and Instrumentation

All reagents including PNIPAM (5500 Da) and cytarabine (AraC) were purchased by Sigma-Aldrich and used as received. Optical absorption UV–vis spectra were acquired by a Perkin Elmer 365 spectrophotometer. Photoluminescence (PL) spectra at different excitation wavelengths were measured by a spectrofluorometer. A quartz cuvette with an optical length of 10 mm was used. NMR spectra were recorded on a Bruker 400 MHz spectrometer at 297 K. Chemical shifts (δ, ppm) are relative to the residual proton solvent peak (H_2_O/D_2_O 4:1 v/v).

### CPDs-PNM Preparation

The CPDs-PNM were prepared from poly(*N*-isopropylacrylamide) (PNIPAM) using an innovative method recently developed in our laboratories.^20^ In detail, an amount of 50 mg of PNIPAM was pyrolyzed for 4 h at 200 °C to obtain a carbon-based material. The dark-reddish solid was washed with 1 mL of Milli-Q water to eliminate the excess of reagent and byproducts and finally sonicated for 2 min. The dispersion was separated by centrifugation (8000 rpm 15 min) and filtration (0.2 μm pore size), and then the red-yellowish supernatant of CPDs-PNM was purified by dialysis using Milli-Q water through a dialysis membrane (10 kDa cutoff) for 46 h.

### Atomic Force Microscopy (AFM)

Imaging was performed in AC mode in air on a commercial AFM instrument (Cypher, Oxford Instruments, Santa Barbara, CA) equipped with a scanner at an XY scan range of 30/40 μm (closed/open loop). Silicon cantilevers (OMCL-AC240TS, ∼ 70 kHz, 2 N/m by Olympus, Japan) were used to acquire scan images in random areas of the samples. To prepare the samples, 10 μL of a colloidal solution of CPDs-PNM (10 mg/mL) was dispensed on freshly cleaved muscovite mica (Ted Pella, Inc., Redding, CA) and left to dry at room temperature in a controlled laboratory environment. AFM images of the height were analyzed using the free tool in MFP-3DTM offline analysis software.

### DLS Measurements

Dynamic light scattering measurements were performed on a ZetaSizer NanoZS90 Malvern Instrument (U.K.), equipped with a 633 nm laser, at a scattering angle of 90° and different temperatures (range 25–40 °C). The size of the particles was calculated from the diffusion coefficient by using the Stokes–Einstein equation.

### Photothermal Measurements

Photothermal properties of CPDs-PNM were investigated by irradiating a glass tube (diameter 3 mm) containing various amounts of the CPDs-PNM dispersion. A volume of 100 μL of the CPDs-PNM dispersion was irradiated with a CW laser 532 nm (different laser power) for various minutes. We used a FLIR infrared thermal imaging camera to measure the temperature of the solution every 20 s, during the heating and cooling processes.

### Drug Loading

To an aqueous solution of CPDs-PNM (17 mg/8 mL), an excess of AraC (1:3 w/w, 51 mg) was added, and the mixture was stirred at rt for 48 h. The sample was subjected to dialysis by a Spectra/Por membrane 10 kDa cut off MWCO upon to absence of drug in the dialysis medium. The amount of AraC entrapped in the carbon dots was measured by optical absorption at 271 nm, referring to a calibration curve.

The drug loading capacity (LC, %) was calculated by the following equation:
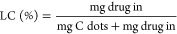
Before the biological assays, the CPDs-PNM/AraC sample was filtered by a 0.2 μm GHP filter. After filtration, the absorbance intensity in the absorption spectrum showed a reduction of 10–13%.

### Photothermal Release of AraC

To assess the photothermal-triggered release of AraC from CPDs-PNM/AraC adducts, aliquots of 600 μL were dispensed on a micromembrane for dialysis (cutoff 3.5–5 kDa) and dipped in 1.7 mL of water in a standard quartz spectroscopic cuvette. The solution was maintained under stirring during all of the experiments. The CPDs-PNM/AraC was exposed to the laser source (532 nm), and the temperature of the CPDs-PNM/AraC dispersion was measured by a thermal camera. The amount of AraC released was spectrophotometrically measured after each laser exposition.

### Computational Methods

The interaction energies of several AraC clusters, henceforth named AraC_*i*_, were evaluated by means of the DFT approach at the B3LYP/6-311G level using the conductor-like polarizable continuum model (CPCM) to assess the solvent effect.^26^ The subscript *i* indicates the number of molecules constituting the cluster and assumes the values of 2, 4, and 6. To simulate experimental conditions, the optimized clusters were placed onto a 15-mer chain of PNIPAM, the adducts PNIPAM-AraC_*i*_ were solvated with water molecules, then 200 ns MD of the solvent was carried out keeping fixed the rest of the system, and a further 200 ns MD simulations were run removing all of the constraints. MD simulations were performed by the Forcite package, under periodic boundary conditions in the NPT ensemble at *P* = 1 atm and a temperature of 310 K. A time step of 1 fs was used to integrate the equation of motion, and the polymer covalent force field was chosen for this purpose. For each adduct, three structures were randomly sampled during the last 10 ns and optimized by the PCFF force field, followed by a PM6 semiempirical approach and, finally, at B3LYP/6-311G level considering the solvent effect by means of the CPCM model. Semiempirical and ab initio calculations were performed by Gaussian 16 software.

### Cell Culture

The human neuroblastoma cell lines, SH-SY5Y, were maintained in DMEM-F12 (Gibco, Thermo Fisher) supplemented with 10% heat-inactivated (HI) fetal bovine serum (Gibco, Thermo Fisher), 100 mg/mL penicillin and streptomycin (Gibco, Thermo Fisher), and 2 mM L-glutamine at 37 °C, 5% CO_2_.

#### AraC Antiproliferative Assay

One day before experiments, 2.5 × 10^4^ cells/well were seeded on a 96-multiwell plate in freshly prepared DMEM-F12 with 5% heat-inactivated (HI) fetal calf serum. Real-time cell proliferation data were collected using the Sartorius Incucyte SX1 system that was placed inside a cell culture incubator. Before treatments, the cells were washed twice with phosphate buffer saline (PBS), and the medium was replaced with freshly prepared DMEM-F12 w/o phenol red supplemented with 5% heat-inactivated (HI) fetal calf serum. The cells were exposed to increasing concentrations of AraC (0.1–1 μM), and plates were incubated for 48 h at 37 °C and 5% CO_2_. Untreated cells were used as a control. Images were collected every 6 h for 48 h, using a ×10 objective. A readout of cytotoxicity over time was obtained by Cell-by-Cell Analysis Software.

### MTT Assay

#### AraC Dose–Response

To test the cytotoxicity of AraC, the cells were seeded at 2.5 × 10^4^ cells/well in a 96-multiwell plate. Before the experiment, the cells were washed twice with phosphate-buffered saline (PBS), and the medium was replaced with freshly prepared DMEM-F12 supplemented with 5% heat-inactivated (HI) fetal calf serum. Then, the cells were exposed to AraC (0.1–5 μM) for 24 h at 37 °C and 5% CO_2_. Untreated cells were used as a control. After 24 h treatment, cell cultures were incubated with MTT (5 mg/mL) for 2 h at 37 °C and then lysed with DMSO. Formazan production was evaluated in a plate reader through the absorbance at 570 nm.

#### CPDs-PNM and CPDs-PNM/AraC Cytotoxicity Test under Light Irradiation

To test the effect of light irradiation on the activity of CPDs-PNM loaded or not with AraC, neuroblastoma cells, SH-SY5Y, were plated at a density of 2.5 × 10^4^ cells/well in 96-multiwell plates. To avoid overheating of bordering wells, the cells were properly spaced. Before the experiment, the cells were washed twice with phosphate-buffered saline (PBS), and the medium was replaced with freshly prepared DMEM-F12 w/o phenol red supplemented with 5% heat-inactivated (HI) fetal calf serum. CPDs-PNM/AraC samples (AraC 10–1 μM range) were prepared by diluting a stock solution of CPDs-PNM (0.2 mg/mL)/AraC (20 μM) with a solution of CPDs-PNM (0.2 mg/mL) to maintain the amount of carbon dots constant. All of the samples were passed through 0.22 μm filters to avoid contaminations; 10 μL of each sample, including CDPs-PNM alone as a control, was added to the cells (90 μL medium) to have a final concentration of 0.02 mg/mL CPDs-PNM and AraC in the 0.1–1 μM range. Each well was individually exposed for 4 min to light irradiation (λ = 532 nm, power density 16.9 W/cm^2^). A treated but nonirradiated plate was taken under the same condition and used as a control. To reduce cellular stress, during light treatment, both multiwells were taken onto heating plates with a preset temperature of 37 °C. During light irradiation, the potential overheating of the well was monitored by a thermal camera. After irradiation, treated and untreated multiwells were placed in the incubator at 37 °C and 5% CO_2_. After 24 h, cell cultures were incubated with MTT (0.5 mg/mL) for 2 h at 37 °C and then lysed with DMSO. Formazan production was evaluated in a plate reader through the absorbance at 570 nm.

### Cellular Uptake Assessment

One day before treatments, 1.2 × 10^5^ cells were seeded onto WillCo-dish Glass Bottom dishes in DMEM-F12 w/o phenol red with 5% fetal calf serum (FCS). Treatments with CPDs-PNM alone and CPDs-PNM loaded with two different concentrations of AraC (0.1 and 1 μM) were used. Untreated cells have been used as a control. After 6 h of exposure, the cells were washed twice with PBS and then fixed in 4% paraformaldehyde in PBS at room temperature for 15 min. Hoechst at a final concentration of 1 μg/mL was added to each dish for specifically staining the nuclei and then washed two times briefly in PBS.

### Laser Scanning Microscope (LSM)

An Olympus FV1000 confocal laser scanning microscope (LSM), equipped with a diode laser (405 nm, 50 mW) and gas lasers (multiline Argon: 457, 488, 515 nm, total 30 mW; HeNe(G): 543 nm, 1 mW and HeNe(R): 633 nm, 1 mW), was used to perform confocal microscopy studies. The detector gain was fixed at a constant value, and images were collected with an oil immersion objective (60× O PLAPO), in sequential mode, randomly all through the area of the well. The following four channels were recorded: λ_ex/em_ = 405/410–430 nm (ch01), λ_ex/em_ = 488/500–530 nm (ch02), λ_ex/em_ = 633/650–700 nm (ch03), and optical bright field (ch04). The image analysis was carried out using Huygens Essential software (by Scientific Volume Imaging B.V., The Netherlands).

## Supporting Information Available

Absorption spectrum of CPDs-PNM at various carbonization process times; ^1^H NMR spectra; modeling simulation data; photothermal conversion efficiency (η) calculation; and optical images of treated cancer cells (PDF)

## Footnotes

a Δ*E*_binding AraC_*i*__ = *E*_electronic AraC_*i*__ – *i*·*E*_electronic AraC_ (*i* = amount of AraC molecules).

b Δ*E*_binding PNIPAM-AraC_*i*__ = *E*_electronic PNIPAM-AraC_*i*__ – *E*_electronic PNIPAM_ – *E*_electronic AraC_*i*__.
