# Supplementary material for: Green Light-Triggerable Chemo-Photothermal Activity of Cytarabine-Loaded Polymer Carbon Dots: Mechanism and Preliminary In Vitro Evaluation
Source: ACS Appl Mater Interfaces. 2023 Jan 23;15(4):5732–43. doi: 10.1021/acsami.2c22500 (PMC9906628; doi:10.1021/acsami.2c22500)
Supplement: Supplementary file 1 — am2c22500_si_001.pdf [file am2c22500_si_001.pdf]

Supporting information

Green Light-Triggerable Chemo-Photothermal Activity of Cytarabine Loaded Polymer Carbon Dots: Mechanism and Preliminary *in vitro* Evaluation

Grazia M. L. Consoli[1,2], Maria Laura Giuffrida[3*], Stefania Zimbone[3], Loredana Ferreri[1], Ludovica Maugeri[4], Michele Palmieri[5], Cristina Satriano[6], Giuseppe Forte[4] and Salvatore Petralia[2,4]**

[1]CNR-Institute of Biomolecular Chemistry, Via Paolo Gaifami 18, 95126 Catania, Italy

[2]CIB-Interuniversity Consortium for Biotechnologies U.O. of Catania, Via Flavia, 23/1, 34148 Trieste, Italy

[3]CNR-Institute of Crystallography, Via Paolo Gaifami 18, 95126 Catania, Italy

[4]Department of Drug Science and Health, University of Catania, Via Santa Sofia 64, 95125 Catania, Italy

[5]CSEM-Swiss Center for Electronics and Microtechnology, Rue Jaquet-Droz 1, 2002 New Chatel, Switzerland

[6]Department of Chemical Science, University of Catania, Via Santa Sofia 64, 95125 Catania, Italy

Content

Figure S1. Optical absorption spectrum of CPDs-PNM dispersion at various carbonization process time.

Figure S2. NMR spectra of CPDs-PNM.

Photothermal conversion efficiency (η)

Figure S3. Photothermal conversion efficiency (η) measurement.

Figure S4. Photothermal experiments for acqueous CPDs-PNM dispersion at 532 nm and different absorbance value.

Figure S5. ^{1}H-NMR spectrum of AraC and CPD-PNM/AraC adduct.

Figure S6. AraC cluster modeling simulation data.

Table S1. Binding energy for PNIPAM-AraC$_i$ adducts.

Figure S7. Representative optical images of not irradiated and irradiated cancer cells treated with CPDs-PNM.

Figure S8. Photothermal effect of CPDs-PNM/AraC dispersion in 96-well plate upon green light irradiation.

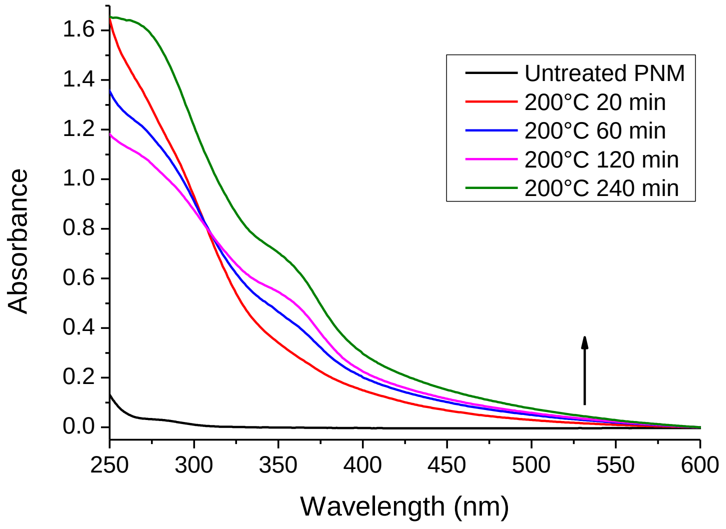

Figure S1. Optical absorption spectrum of CPDs-PNM dispersion at various carbonization process time.

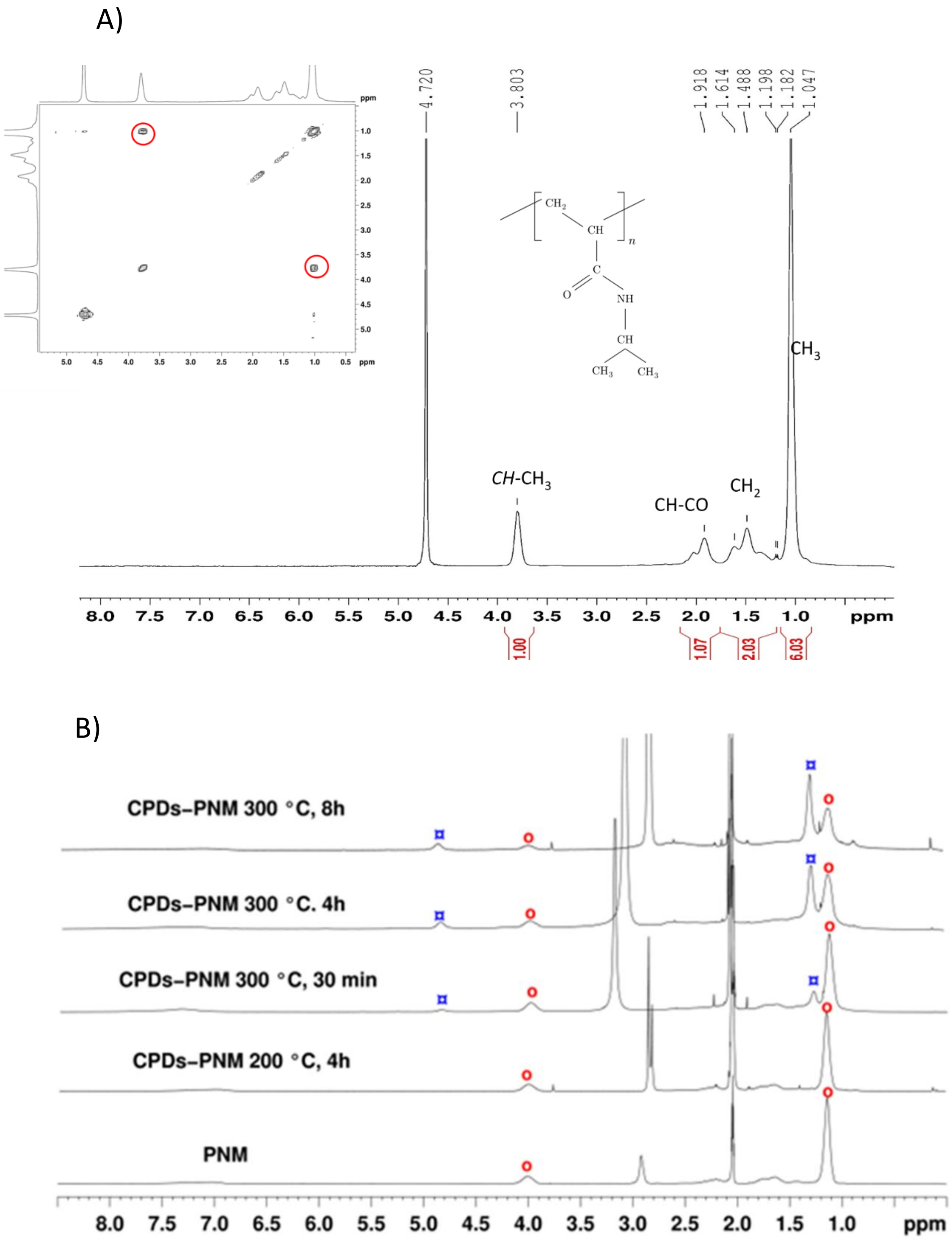

Figure S2. A) ^{1}H-NMR spectrum and 2D-COSY NMR spectrum (inset) of CPDs-PNM prepared at 200 °C, 4h (D_2O, 400.13 MHz, 297 K); **B)** ^{1}H-NMR spectra (Acetone-*d6*, 400.13 MHz, 297 K) of CPDs-PNM prepared by heating at different temperature and time. At lower temperature (200 °C) only the iso-propyl signals of the PNIPAM (abbreviated to PNM) pendants are visible (red circles). A novel pattern of signals, related to the iso-propyl groups bonded to aromatic nitrogen atoms (blue squares), appears at 300 °C and the signal intensity increase prolonging the reaction time from 30 min to 8 h.

Photothermal conversion efficiency (η) calculation

Photothermal measurements were performed irradiating for 10 minutes a glass tube (diameter 3 mm) containing a volume of 100 μL of CPDs-PNM dispersion (2 mg mL^{-1}), using a continuous wave Laser 532 nm (power 300 mW). A Flirck infrared thermal imaging camera was used to easure the temperature of solution every 10 seconds, during the heating and cooling processes. The photothermal conversion efficiency (η) was calculated according to equation (1) introduced by Roper et al. [Roper, D. K.; Ahn, W.; Hoepfner, M. *J. Phys. Chem. C* **2007**, *111*(9), 3636-3641].

$$\eta = \frac{hA\,(T_{max} - T_{surr}) - Q_{Dis}}{I(1-10^{-A})} \quad (1)$$

where T_{max} (38.0 °C) and T_{surr} (21.7 °C) represents the max photothermal temperature and

the ambient temperature respectively. The absorbance (A) of CNPs-PNIPAM at 532 nm and I is the incident laser power (W). Then, equations (2) and (3) were introduced to obtain unknow hA.

$$\theta = \frac{T - T_{surr}}{T_{max} - T_{surr}} \quad (2)$$

$$\tau = \frac{M_D\, C_D}{hA}1 \quad (3)$$

where M_D and C_D are the mass of water (0.1 g) and heat capacity (4.2 J g^{-1}) of water, respectively, and τ_s is the time constant. τ_s was experimentally calculated by the equation (4).

$$t = -\tau(ln\theta) \quad (4)$$

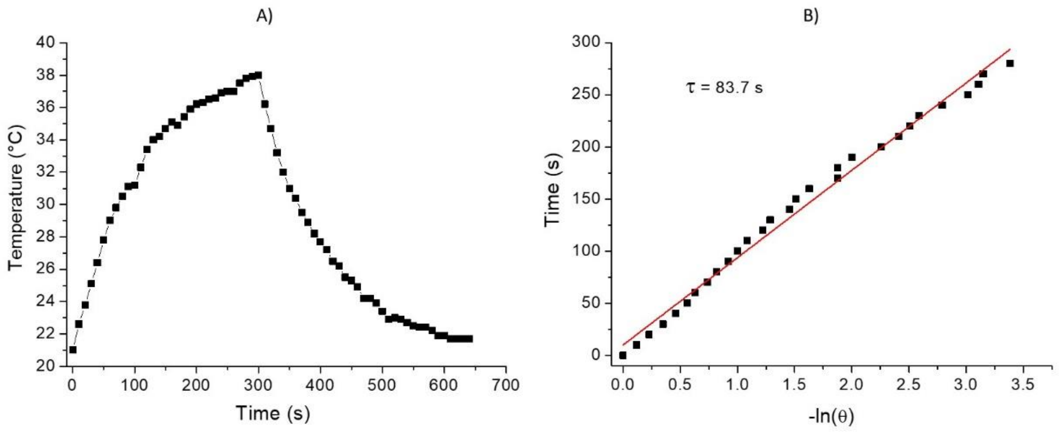

Figure S3. Photothermal conversion efficiency (η) measurement: A) photothermal effect of CPDs-PNM dispersion (100 μL, Ass532nm= 0.43 and B) linear relationship between time (sec) and -ln(θ), (the slope is τs).

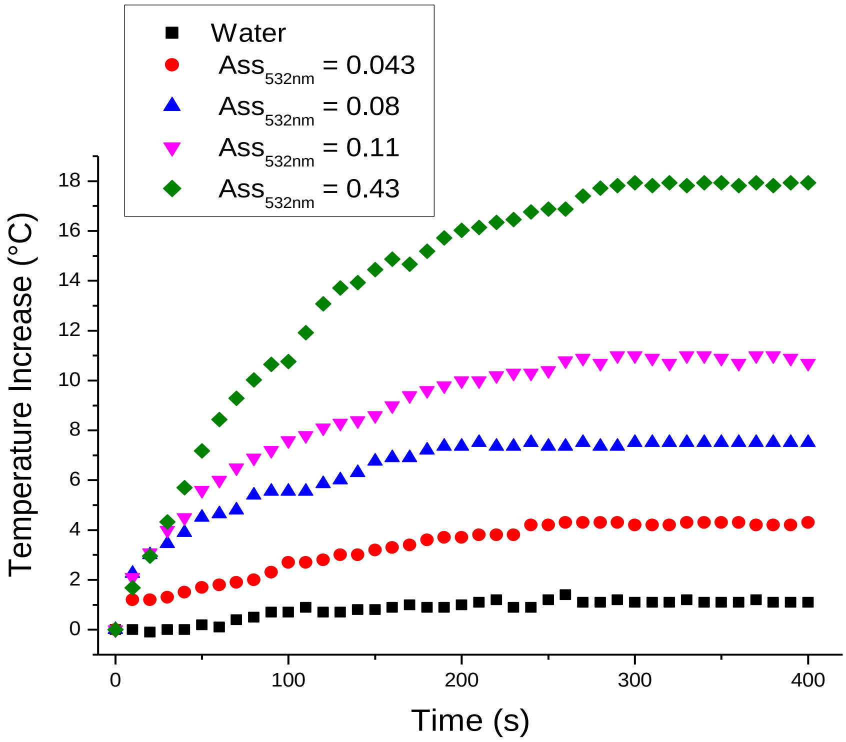

Figure S4. Photothermal experiments for acqueous CPDs-PNM dispersion at different absorbance value at 532 nm (Laser power density 16.9 W cm^{-2}).

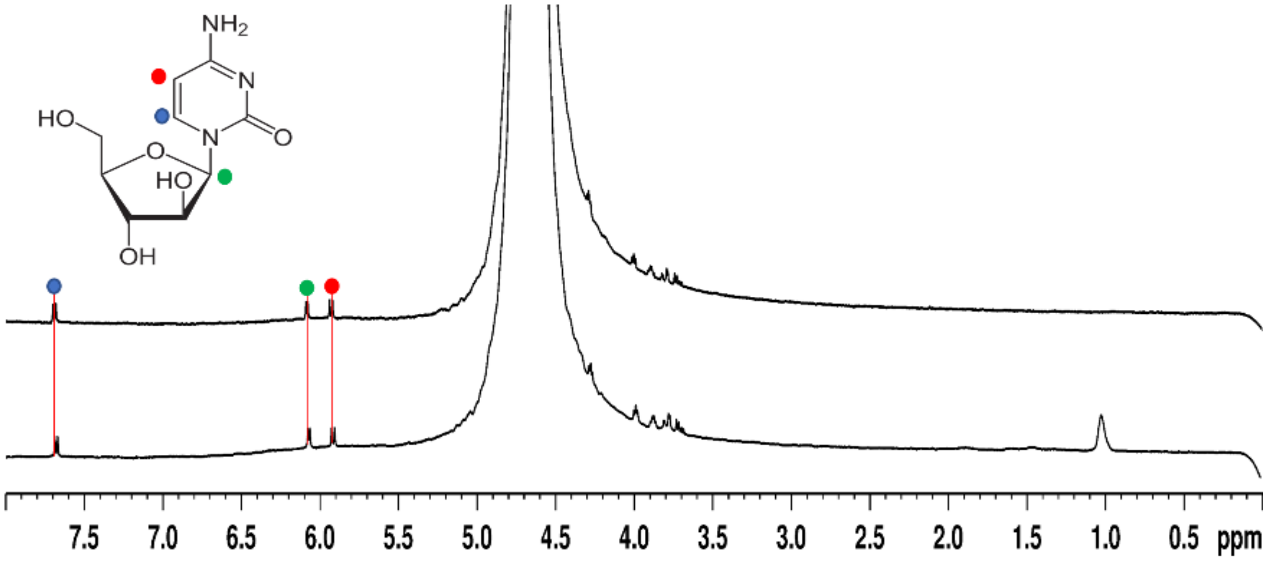

Figure S5. ^{1}H-NMR spectra of AraC (up) and CPD-PNM/AraC adduct (bottom) (400.13 MHz, H_2O:D_2O 4:1 *v/v*, 297 K). Upfield shift of the Ara-C pyrimidine ring CH protons (doublets at 5.93 ppm and 7.7 ppm; $\Delta\delta$ 0.013 and 0.012 respectively) and sugar CH proton (doublet at 6.08 ppm, $\Delta\delta$ 0.012).

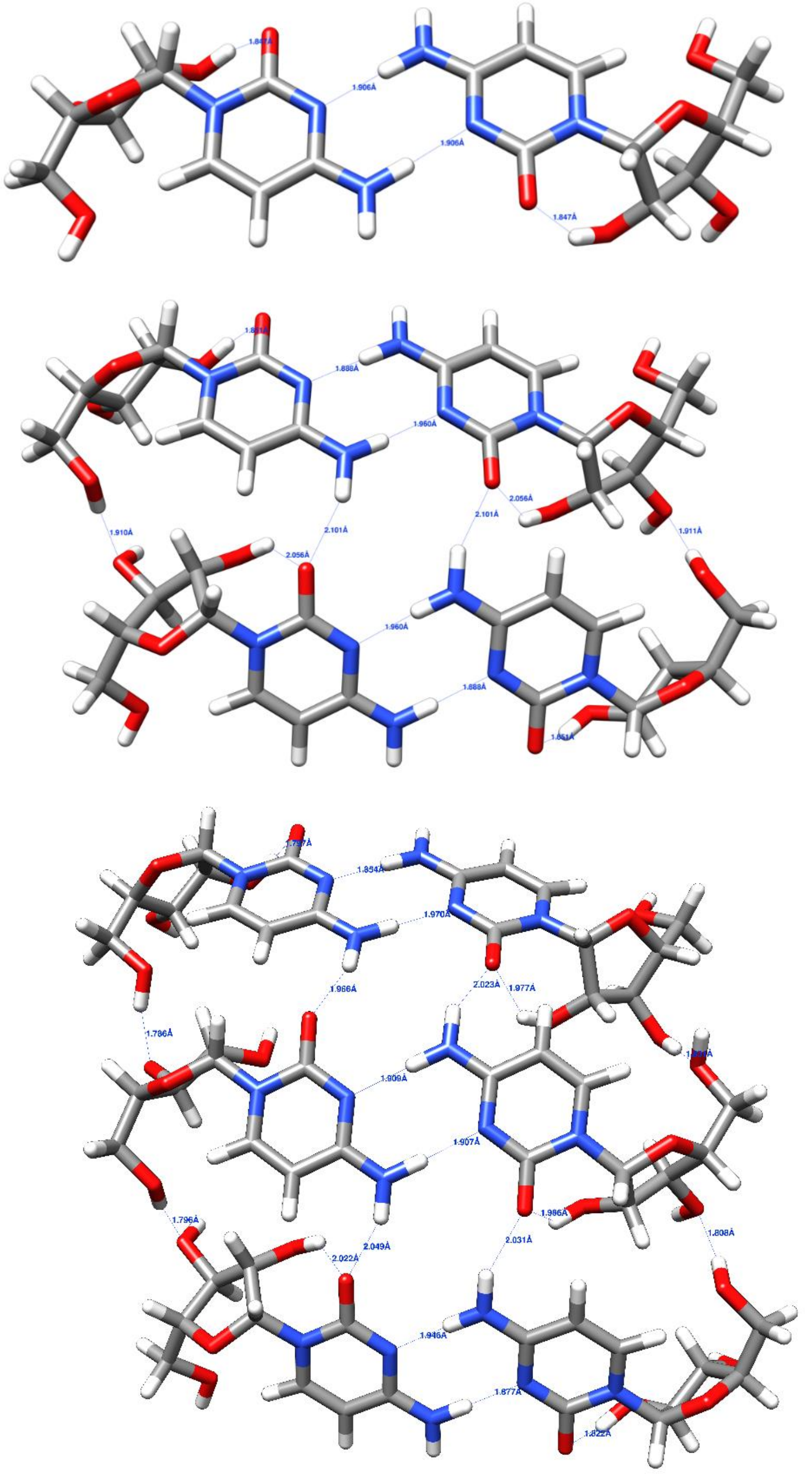

Figure S6. AraC cluster modelling simulation data: clusters of AraC with 2, 4 and 6 molecules of AraC, the hydrogen bonds (blue line) and H-bond lengths are shown.

Table S1. Binding energy for the PNIPAM-AraCi adducts are reported for the three sampled structures. The negative Energy binding values for the PNIPMAN-ARAC(i) adducts indicate a favourable interaction in the cases of i = 1, 2 and 4, while increasing the AraC molecules to 6 units the formation of the adduct seems to be thermodynamically unfavourited. It could be explained by the small size of the polymer chain and an extension to 20-25-mer would result in a more effective loading of AraC molecules.

Replicas	$E_{PNIPAM\text{-}AraC1} - E_{AraC1} - E_{PNIPAM}$ (kcal/mol)	$E_{PNIPAM\text{-}AraC2} - E_{AraC2} - E_{PNIPAM}$ (kcal/mol)	$E_{PNIPAM\text{-}AraC4} - E_{AraC4} - E_{PNIPAM}$ (kcal/mol)
1	**-15.59**	-17.11	**-19.34**
2	-12.78	-14.42	-17.33
3	-14.24	**-17.63**	-19.11

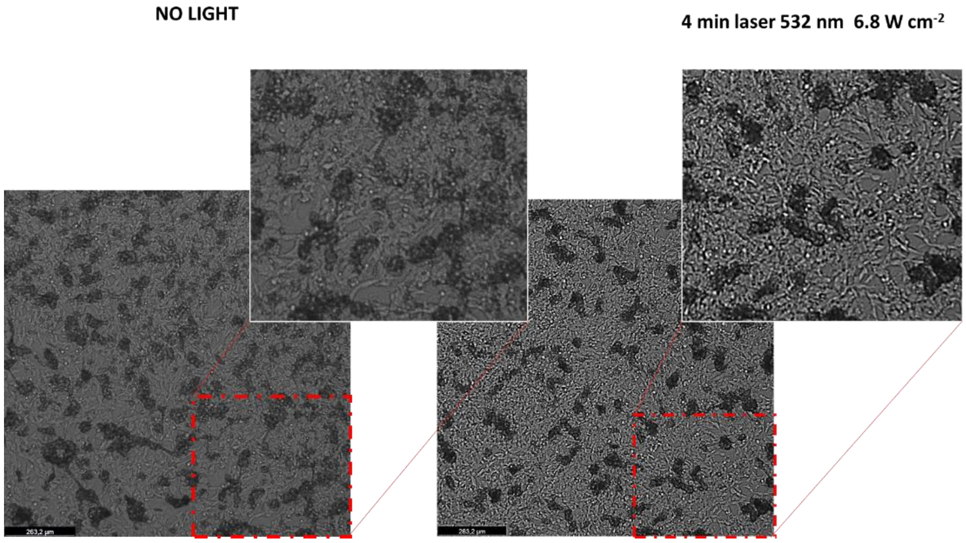

Figure S7. Representative optical images of not irradiated and irradiated cancer cells treated with CPDs-PNM. Images related to the quantitative results depicted in Figure 9B in the manuscript. No cells damage was observed upon light exposure.

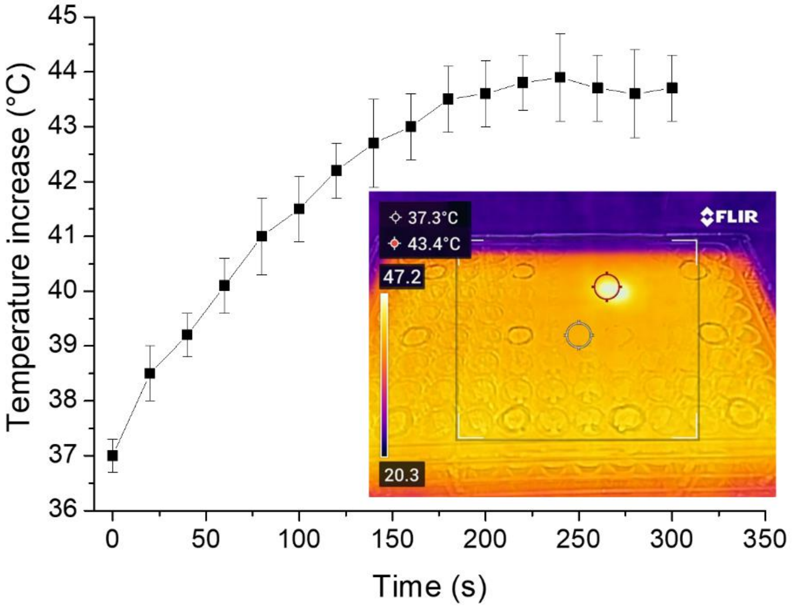

Figure S8. Photothermal effect of CPDs-PNM/AraC dispersion (100 μL, CPD, A_{532nm}= 0.07) in 96-well plate upon green light irradiation (16.9 W cm^{-2}).
